# Crystal structure and Hirshfeld-surface analysis of the pesticide etoxazole

**DOI:** 10.1107/S2056989025001173

**Published:** 2025-02-18

**Authors:** Chaluvarangaiah Sowbhagya, Thaluru M. Mohan Kumar, Hemmige S. Yathirajan, Sean Parkin

**Affiliations:** a Department of Physical Sciences, Amrita School of Engineering, Amrita Vishwa Vidyapeetham, Bengaluru-560 035, India; b Department of Studies in Chemistry University of Mysore, Manasagangotri Mysuru-570 006 India; c Department of Chemistry University of Kentucky,Lexington KY 40506-0055 USA; Institute of Chemistry, Chinese Academy of Sciences

**Keywords:** etoxazole, insecticide, acaricide, Hirshfeld-surface analysis, crystal structure

## Abstract

Etoxazole (C_21_H_23_F_2_NO_2_), systematic name 4-(4-*tert*-butyl-2-eth­oxy­phen­yl)-2-(2,6-di­fluoro­phen­yl)-4,5-di­hydro-1,3-oxazole, is a fluorinated insecticide and acaricide that inhibits chitin biosynthesis, disrupting insect development by preventing proper exoskeleton formation. Widely used in agriculture since 1998, it is readily absorbed by plant tissues and translocates within leaves. Metabolic studies have identified several oxidative degradation products, while toxicol­ogical assessments have examined potential effects, including oxidative stress. This study presents a detailed crystallographic and Hirshfeld surface analysis of etoxazole. The mol­ecule consists of a central di­hydro-oxazole ring flanked by 2,6-di­fluoro­phenyl and 4-*tert*-butyl-2-eth­oxy­phenyl groups, each twisted relative to the oxazole core. The di­hydro-oxazole ring is nearly planar, with the substituted phenyl rings forming dihedral angles of 44.20 (4)° and 47.87 (4)° with the mean plane of the di­hydro-oxazole. The eth­oxy group exhibits a dihedral angle of 15.04 (11)° to the *tert*-butyl­phenyl ring, while the *tert*-butyl group itself shows minor torsional disorder [major:minor occupancies are 0.760 (6):0.240 (6)]. The mol­ecular packing is dominated by van der Waals-type inter­actions, though weak C—H⋯F and C—H⋯O inter­actions lead to pleated layers parallel to the *ab* plane, which further stack along the *c*-axis direction. A Hirshfeld surface analysis confirms the prevalence of van der Waals inter­actions in crystal stabilization.

## Abstract 2

The crystal structure of the insecticide/acaricide etoxazole is presented along with a Hirshfeld surface analysis of inter­molecular inter­actions present in the crystal structure.

## 1. Chemical context

Etoxazole is a fluorinated insecticide and acaricide that has been widely utilized in agriculture since its introduction in 1998 (Park *et al.*, 2020 ▸). As a member of the oxazoline class, it disrupts insect development by inhibiting chitin biosynthesis, a mechanism that prevents the proper formation of the exo­skeleton. Etoxazole is readily absorbed by plant tissues, where it undergoes limited translocation within leaves. Its effectiveness and chemical properties have been extensively studied, with comprehensive reviews available on the biological activities of oxazole derivatives (Kakkar & Narasimhan, 2019 ▸) and their synthetic methodologies (Joshi *et al.*, 2023 ▸). Concerns regarding its potential toxic effects, including oxidative stress, have also been explored in recent toxicol­ogical assessments (Macar *et al.*, 2022 ▸). Metabolic studies have identified several degradation products of etoxazole, which arise primarily through oxidative transformations. These metabolites have been detected in environmental and biological systems using high-resolution analytical techniques (Sun *et al.*, 2019 ▸). Among these, oxidation at the oxazole ring leads to the formation of its metabolite ‘R13’ (APVMA, 2024 ▸; Mohan Kumar *et al.*, 2024 ▸). Previous structural studies have examined various insecticidal compounds, including phenyl­pyrazole derivatives (Priyanka *et al.*, 2022 ▸; Vinaya *et al.*, 2023 ▸), inter­mediates involved in anthranilamide synthesis (Lei *et al.*, 2009 ▸), and other oxazole-containing insecticides such as ethyl 3-(4-chloro­phen­yl)-5-[(*E*)-2-(di­methyl­amino)­ethen­yl]-1,2-oxa­zole-4-carboxyl­ate (Efimov *et al.*, 2015 ▸). Additionally, the crystal structure of fipronil, another important insecticide, has been reported (Park *et al.*, 2017 ▸). Recognizing the significance of etoxazole in pest management, this study provides a detailed crystallographic analysis and Hirshfeld surface investigation of its mol­ecular and crystal structure. Understanding its conformation and inter­molecular inter­actions offers valuable insights into its stability, physicochemical behaviour, and potential reactivity.
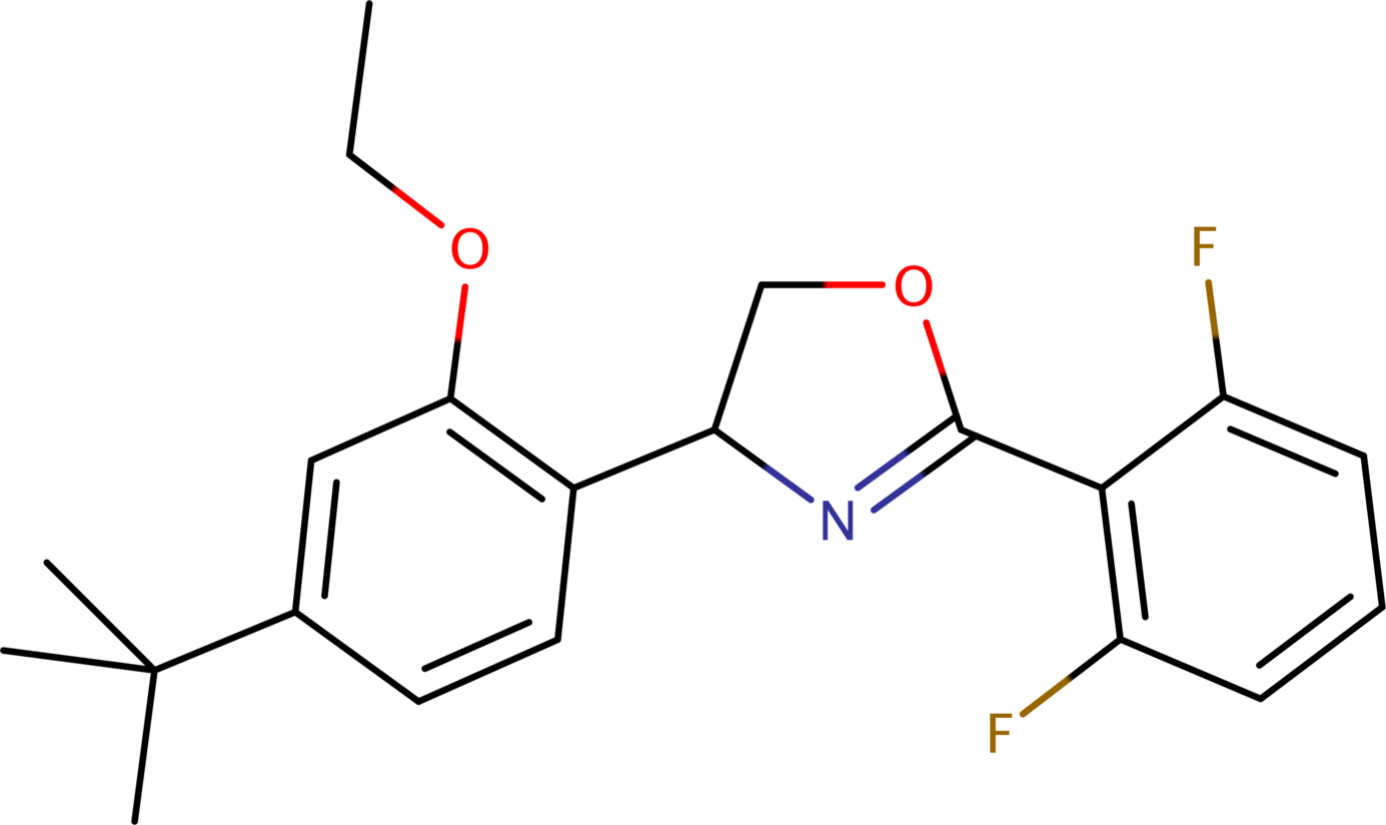


## 2. Structural commentary

**Figure 1:**
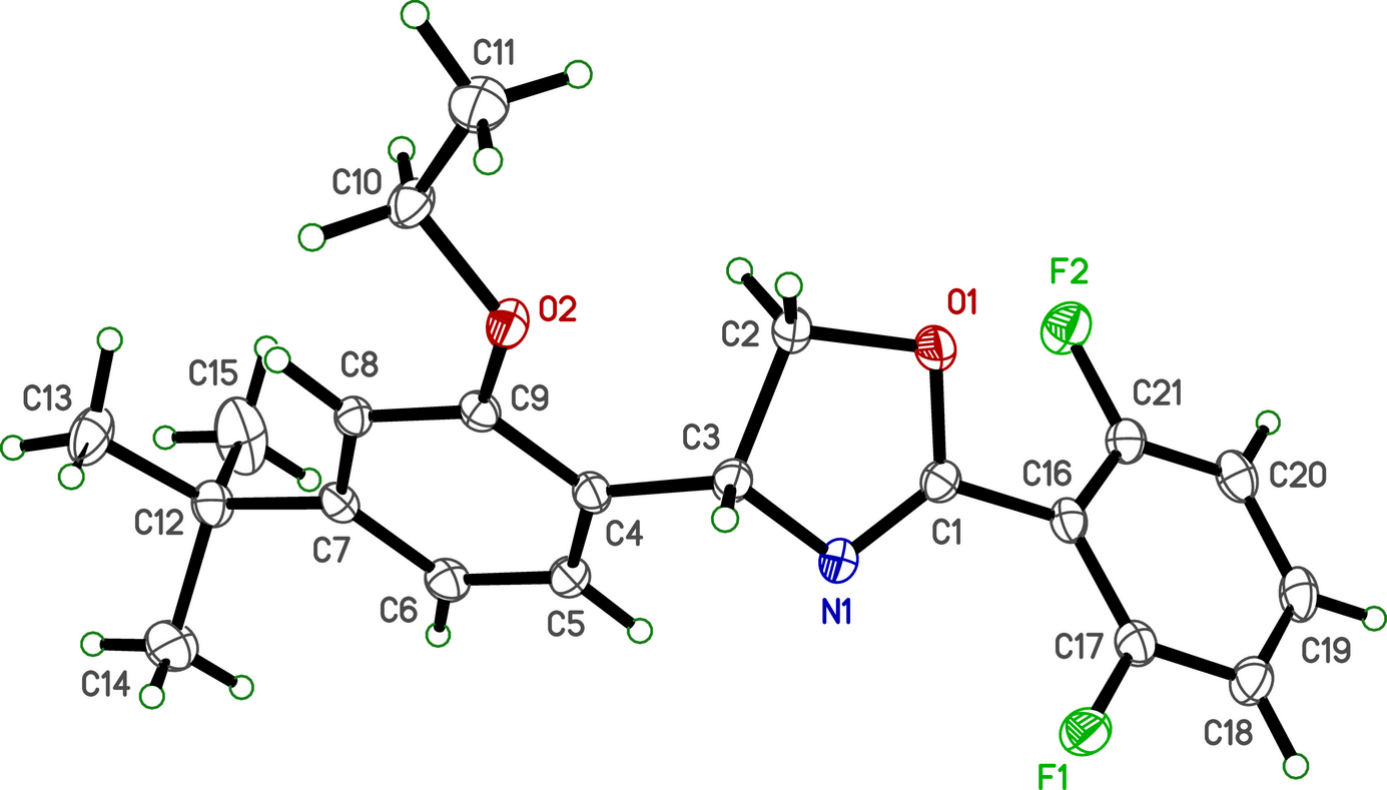
An ellipsoid plot (50% probability) of etoxazole. For the sake of clarity, only the major component of disorder for the *tert*-butyl group is shown. Hydrogen atoms are drawn as small arbitrary circles.

The crystal structure of etoxazole is monoclinic, space group type *P*2_1_/*n*. The mol­ecule (Fig. 1 ▸) is comprised of three rings: a 4,5-di­hydro-1,3-oxazole heterocycle with a 2,6-di-fluoro­phenyl group attached to C1 (between N1 and O1 of the oxazole) and a 4-*tert*-butyl-2-eth­oxy­phenyl group bonded to C3, adjacent to N1 on the opposite side from C1. The di­hydro-oxazole ring is only very slightly puckered; its r.m.s. deviation from planarity is 0.0514 Å with a maximum deviation of 0.0695 (6) Å at C2. All bond lengths and angles are within normal ranges.

The mol­ecular conformation is a consequence of the twist of each substituted phenyl ring to the central heterocyclic ring. The dihedral angles between the mean plane of the di­hydro-oxazole ring and the attached di-fluoro­phenyl (atoms C16–C21) and 4-*tert*-butyl-2-eth­oxy­phenyl (atoms C4–C9) are 44.20 (4)° and 47.87 (4)°, respectively, and by the torsion angles C5—C4—C3—N1 = 6.09 (14)° and C5—C4—C3—C2 = 124.39 (11)°. The orientation of the eth­oxy group (O2—C10—C11) relative to the 4-*tert*-butyl­phenyl ring gives a dihedral angle of 15.04 (11)° and torsion C8—C9—O2—C10 = −17.70 (14)°. Lastly, the *tert*-butyl group is torsionally disordered over two positions with refined occupancy factors of 0.760 (6) and 0.240 (6). The angular deviation of minor to major components is 23.0 (3)°, calculated as the weighted mean of the differences between torsion angles of the form C6—C7—C12—C13,14,15 and C6—C7—C12—C13′,14′,15′.

## 3. Supra­molecular features

**Figure 2:**
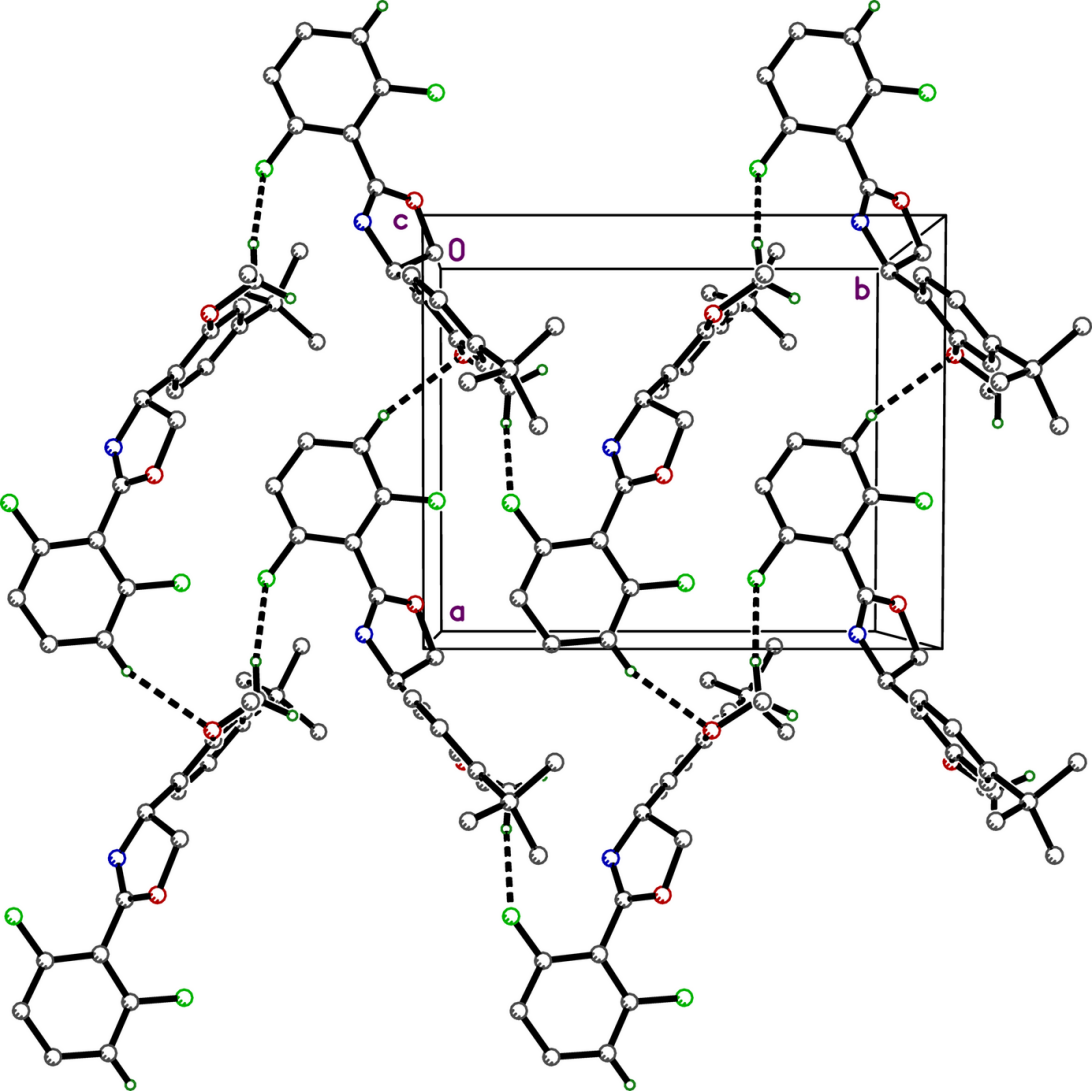
A partial packing plot viewed normal to the *ab*-plane, showing weak C—H⋯F and C—H⋯O contacts that connect the mol­ecules into pleated layers.

**Figure 3:**
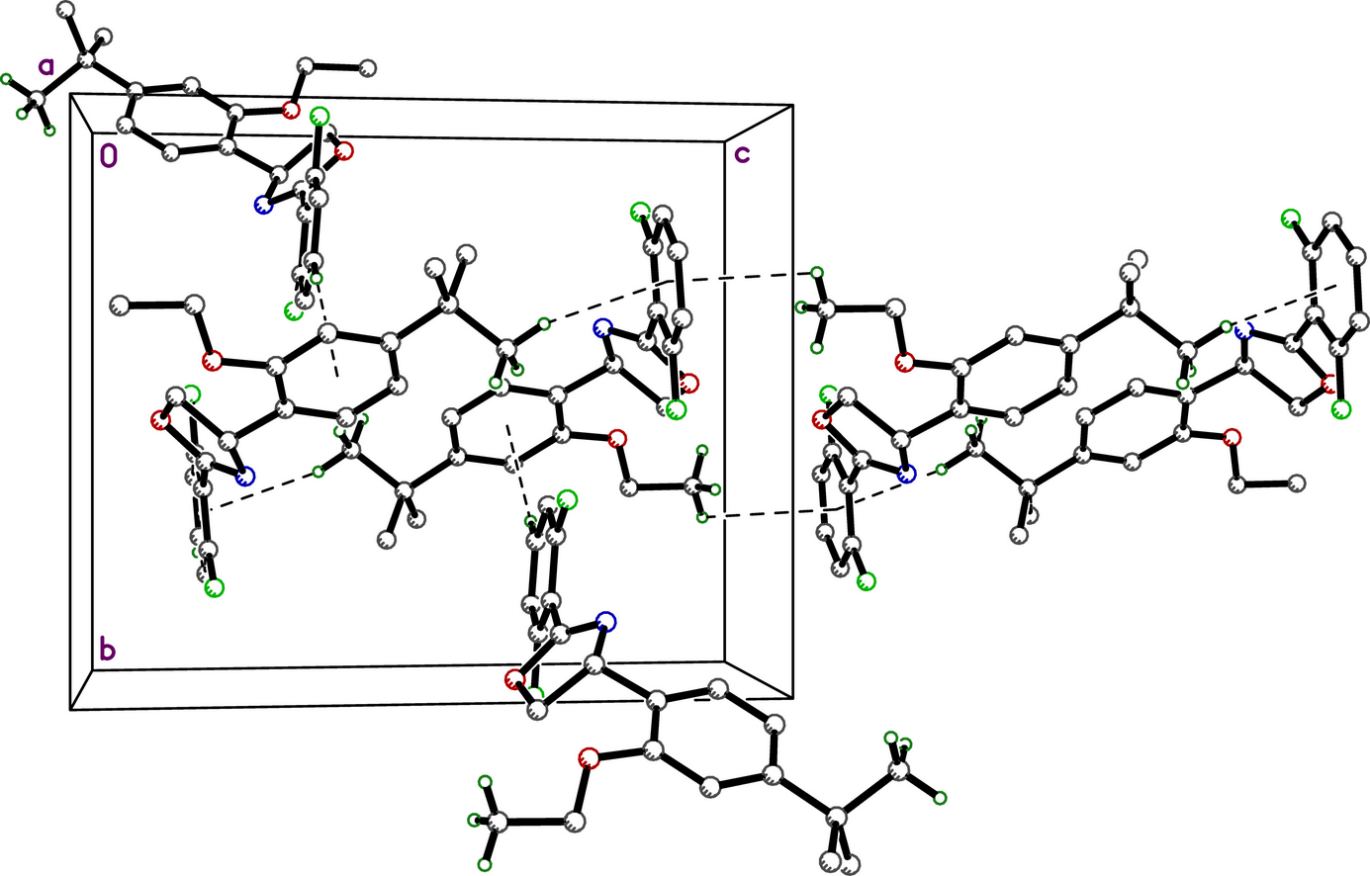
A partial packing plot viewed down the *a*-axis showing weak C—H⋯π inter­actions that connect pleated layers of mol­ecules (Fig. 2 ▸) to generate the full three-dimensional structure.

There are no strong hydrogen bonds in the crystal structure of etoxazole. Suggestions for ‘potential hydrogen bonds’ provided by *SHELXL* (Sheldrick, 2015*b* ▸) and by *Mercury* (Macrae *et al.*, 2020 ▸), however, flag two close contacts: C10—H10*A*⋯F1^i^ [*d_D-A_* =- 3.5211 (13) Å; symmetry code: (i) −*x* + 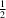, *y* + 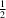, −*z* + 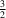] and C20—H20⋯O2^ii^ [*d*(*D*⋯*A*) = 3.4766 (13) Å; symmetry code: (ii) *x* + 1, *y*, *z*] (Table 1 ▸), which together weakly link the mol­ecules into diperiodic pleated layers parallel to the *ab* plane (Fig. 2 ▸). There are no π–π stacking inter­actions, but there are several C—H⋯π contacts: C19—H19⋯*Cg*(C4–C9)^iii^ [*d*(H⋯*A*) = 3.5301 Å; symmetry code: (iii) −*x* + 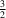, *y* − 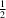, −*z* + 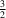] connects 2_1_-screw related mol­ecules; pairs of mutual contacts between mol­ecules of the form C14—H14*A*⋯*Cg(*C16–C21)^iv^ [*d*(*D*⋯*A*) = 2.9092 Å; symmetry code: (iv) −*x* + 1, −*y* + 1, −*z* + 1) combine to form inversion-related pairs; lastly, the methyl group at C11 of the *tert*-butyl ligand closely abuts an inversion-related di­fluoro­phenyl ring C11—H11*A*,*B*,*C*⋯*Cg*^v^ [*d*(*D*⋯*A*) = 3.1319, 3.2496, 3.4314 Å for H11*A*, H11*B*, H11*C*, respectively; symmetry code: (v) −*x* + 1, −*y* + 1, −*z* + 2]. In combination, these contacts (Fig. 3 ▸) stack the pleated layers along the *c*-axis direction, giving rise to the overall 3D structure.

**Figure 4:**
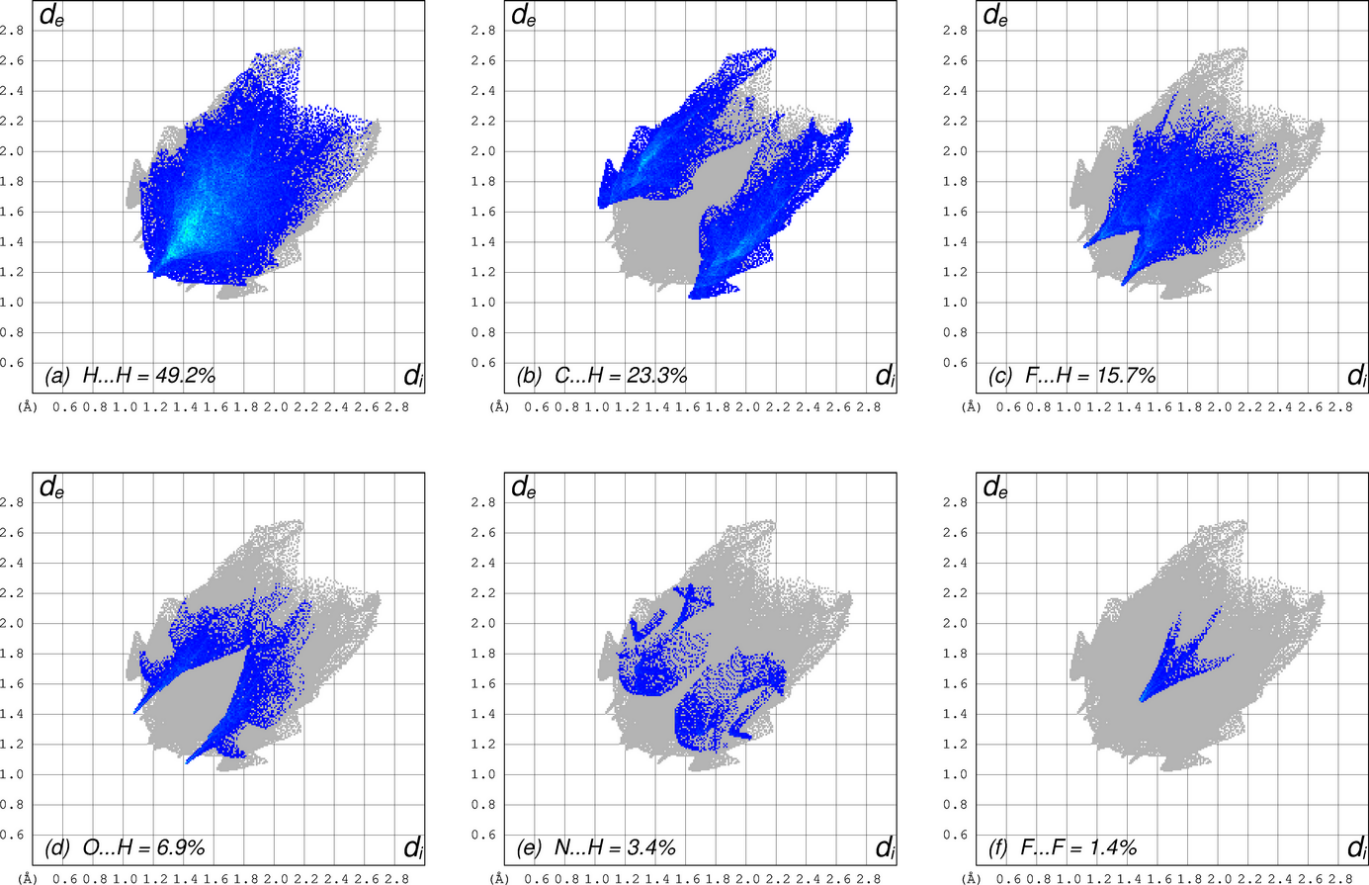
Two-dimensional fingerprint plots qu­anti­fying the various atom–atom contact coverages present in the crystal packing: (*a*) H⋯H = 49.2%; (*b*) C⋯H = 23.3%; (*c*) F⋯H = 15.7%; (*d*) O⋯H = 6.9%; (*e*) N⋯H = 3.4%; (*f*) F⋯F = 1.4%

A Hirshfeld surface analysis (minor disorder component excluded) using *CrystalExplorer* (Spackman *et al.*, 2021 ▸) indicates that almost all (98.6%) inter­molecular contacts involve hydrogen, with the vast majority being H⋯H (49.2%) and C⋯H (23.3%) contacts. Thus, van der Waals inter­actions are particularly prominent in the crystal structure. The full set of inter­molecular inter­actions are summarized as Hirshfeld surface contact fingerprint plots in Fig. 4 ▸.

## 4. Database survey

Given the structural similarity between etoxazole and its R13 metabolite, a previous database survey (CSD v5.45, with updates as of March 2024; Groom *et al.*, 2016 ▸) conducted for the R13 metabolite (Mohan Kumar *et al.*, 2024 ▸) is also applicable to etoxazole itself. That search used a mol­ecular fragment consisting of the three-ring backbone, with the fluorine, eth­oxy, and *tert*-butyl substituents removed, and the oxazole ring’s double bonds set to ‘any type of bond’ in order to capture both oxazole and di­hydro-oxazole variants; the search generated 336 hits. A similar search retaining both fluorine atoms returned only two matches: DOGMEV (Roque *et al.*, 2023 ▸) and LIYZUS (Saha *et al.*, 2023 ▸). As of version 5.46 of the CSD (Nov. 2024), the R13 metabolite is also included in the database as UGUQUM (Mohan Kumar *et al.*, 2024 ▸).

## 5. Synthesis and crystallization

The sample of etoxazole was provided as a gift by Honeychem Pharma Research, India. It was purified by column chromatography and recrystallized from hexane by slow evaporation to obtain clear colourless crystals (m.p.: 375 K).

## 6. Refinement

Crystal data, data collection, and structure refinement details are provided in Table 2 ▸. All full occupancy and major disorder component hydrogens were present in difference-Fourier maps, but were subsequently included in the refinement using riding models, with constrained distances of 0.95 Å (*R*_2_CH), 0.99 Å (*R*_2_CH_2_) and 0.98 Å (*R*CH_3_). *U*_iso_(H) parameters were set to either 1.2*U*_eq_ or 1.5*U*_eq_ (*R*CH_3_ only) of the attached carbon. Two-component torsional disorder of the *tert*-butyl group was handled as separate PARTs [major:minor = 0.760 (6):0.240 (6)] with EADP constraints and SAME geometry restraints included to ensure stable refinement.

## Supplementary Material

Crystal structure: contains datablock(s) I, global. DOI: 10.1107/S2056989025001173/nx2020sup1.cif

Structure factors: contains datablock(s) I. DOI: 10.1107/S2056989025001173/nx2020Isup2.hkl

Supporting information file. DOI: 10.1107/S2056989025001173/nx2020Isup3.cml

CCDC reference: 2422554

Additional supporting information:  crystallographic information; 3D view; checkCIF report

## Figures and Tables

**Table 1:** Close contacts (Å, °) in crystalline etoxazole

*D*—H⋯*A*	*D*—H	H⋯*A*	*D*⋯*A*	*D*—H⋯*A*
C10—H10*A*⋯F1^i^	0.99	2.57	3.5211 (13)	162.1
C20—H20⋯O2^ii^	0.95	2.60	3.4766 (13)	153.9
C—H⋯centroid^*a*^				
C19—H19⋯*Cg*(C4–C9)^iii^			3.5301	
C14—H14*A*⋯*Cg*(C16–C21)^iv^			2.9092	
C11—H11*A*⋯*Cg*^v^			3.1319	
C11—H11*B*⋯*Cg*^v^			3.2496	
C11—H11*C*⋯*Cg*^v^			3.4314	

Symmetry codes: (i) −*x* + 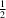, *y* + 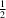, −*z* + 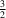; (ii) *x* + 1, *y*, *z*; (iii) −*x* + 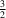, *y* − 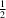, −*z* + 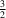; (iv) −*x* + 1, −*y* + 1, −*z* + 1; (v) −*x* + 1, −*y* + 1, −*z* + 2.

**Table 2:** Experimental details

Crystal data	
Chemical formula	C_21_H_23_F_2_NO_2_
*M*_r_	359.40
Crystal system, space group	Monoclinic, *P*2_1_/*n*
Temperature (K)	100
*a*, *b*, *c* (Å)	10.2254 (2), 12.2767 (3), 14.7404 (3)
β (°)	93.726 (1)
*V* (Å^3^)	1846.51 (7)
*Z*	4
Radiation type	Mo *K*α
μ (mm^−1^)	0.10
Crystal size (mm)	0.30 × 0.29 × 0.24

Data collection	
Diffractometer	Bruker D8 Venture dual source
Absorption correction	Multi-scan (*SADABS*; Krause *et al.*, 2015 ▸)
*T*_min_, *T*_max_	0.913, 0.971
No. of measured, independent and observed [*I* > 2σ(*I*)] reflections	36219, 4232, 3810
*R*_int_	0.028
(sin θ/λ)_max_ (Å^−1^)	0.651

Refinement	
*R*[*F*^2^ > 2σ(*F*^2^)], *wR*(*F*^2^), *S*	0.032, 0.077, 1.07
No. of reflections	4232
No. of parameters	249
No. of restraints	6
H-atom treatment	H-atom parameters constrained
Δρ_max_, Δρ_min_ (e Å^−3^)	0.27, −0.17

Computer programs: *APEX5* (Bruker-AXS, 2023 ▸), *SHELXT* (Sheldrick, 2015*a* ▸), *SHELXL2019/3* (Sheldrick, 2015*b* ▸), *XP* in *SHELXTL* (Sheldrick, 2008 ▸), *SHELX* (Sheldrick, 2008 ▸) and *publCIF* (Westrip, 2010 ▸).

## supplementary crystallographic information

### 4-(4-tert-Butyl-2-ethoxyphenyl)-2-(2,6-difluorophenyl)-4,5-dihydro-1,3-oxazole Crystal data

**Table:**

C_21_H_23_F_2_NO_2_	*F*(000) = 760
*M**_r_* = 359.40	*D*_x_ = 1.293 Mg m^−^^3^
Monoclinic, *P*2_1_/*n*	Mo *K*α radiation, λ = 0.71073 Å
*a* = 10.2254 (2) Å	Cell parameters from 9926 reflections
*b* = 12.2767 (3) Å	θ = 2.4–27.6°
*c* = 14.7404 (3) Å	µ = 0.10 mm^−^^1^
β = 93.726 (1)°	*T* = 100 K
*V* = 1846.51 (7) Å^3^	Solvent-rounded block, colourless
*Z* = 4	0.30 × 0.29 × 0.24 mm

### 4-(4-tert-Butyl-2-ethoxyphenyl)-2-(2,6-difluorophenyl)-4,5-dihydro-1,3-oxazole Data collection

**Table:**

Bruker D8 Venture dual source diffractometer	4232 independent reflections
Radiation source: microsource	3810 reflections with *I* > 2σ(*I*)
Detector resolution: 7.41 pixels mm^-1^	*R*_int_ = 0.028
φ and ω scans	θ_max_ = 27.6°, θ_min_ = 2.2°
Absorption correction: multi-scan (*SADABS*; Krause *et al*., 2015)	*h* = −13→12
*T*_min_ = 0.913, *T*_max_ = 0.971	*k* = −15→15
36219 measured reflections	*l* = −19→19

### 4-(4-tert-Butyl-2-ethoxyphenyl)-2-(2,6-difluorophenyl)-4,5-dihydro-1,3-oxazole Refinement

**Table:**

Refinement on *F*^2^	Primary atom site location: structure-invariant direct methods
Least-squares matrix: full	Secondary atom site location: difference Fourier map
*R*[*F*^2^ > 2σ(*F*^2^)] = 0.032	Hydrogen site location: difference Fourier map
*wR*(*F*^2^) = 0.077	H-atom parameters constrained
*S* = 1.07	*w* = 1/[σ^2^(*F*_o_^2^) + (0.0229*P*)^2^ + 0.7589*P*] where *P* = (*F*_o_^2^ + 2*F*_c_^2^)/3
4232 reflections	(Δ/σ)_max_ = 0.001
249 parameters	Δρ_max_ = 0.27 e Å^−^^3^
6 restraints	Δρ_min_ = −0.17 e Å^−^^3^

### 4-(4-tert-Butyl-2-ethoxyphenyl)-2-(2,6-difluorophenyl)-4,5-dihydro-1,3-oxazole Special details

**Table:**

Geometry. All esds (except the esd in the dihedral angle between two l.s. planes) are estimated using the full covariance matrix. The cell esds are taken into account individually in the estimation of esds in distances, angles and torsion angles; correlations between esds in cell parameters are only used when they are defined by crystal symmetry. An approximate (isotropic) treatment of cell esds is used for estimating esds involving l.s. planes.

### 4-(4-tert-Butyl-2-ethoxyphenyl)-2-(2,6-difluorophenyl)-4,5-dihydro-1,3-oxazole Fractional atomic coordinates and isotropic or equivalent isotropic displacement parameters (Å^2^)

**Table:**

	*x*	*y*	*z*	*U*_iso_*/*U*_eq_	Occ. (<1)
F1	0.65801 (6)	0.16709 (5)	0.81007 (5)	0.02591 (15)	
F2	0.84932 (6)	0.51354 (5)	0.84668 (5)	0.02856 (16)	
O1	0.59310 (7)	0.46735 (6)	0.88831 (5)	0.02189 (17)	
O2	0.20619 (7)	0.56908 (6)	0.80997 (5)	0.01919 (16)	
N1	0.52318 (8)	0.36866 (7)	0.76447 (6)	0.01943 (18)	
C1	0.61498 (10)	0.39273 (8)	0.82283 (6)	0.01647 (19)	
C2	0.46203 (10)	0.50925 (9)	0.86463 (7)	0.0203 (2)	
H2A	0.465680	0.585120	0.842192	0.024*	
H2B	0.407431	0.507000	0.917657	0.024*	
C3	0.40756 (10)	0.43176 (8)	0.78894 (7)	0.0178 (2)	
H3	0.343741	0.380819	0.815230	0.021*	
C4	0.34021 (9)	0.48827 (8)	0.70751 (7)	0.01653 (19)	
C5	0.37576 (10)	0.47310 (8)	0.61912 (7)	0.0193 (2)	
H5	0.444699	0.424052	0.607837	0.023*	
C6	0.31205 (10)	0.52859 (9)	0.54676 (7)	0.0202 (2)	
H6	0.338574	0.516894	0.486953	0.024*	
C7	0.21004 (10)	0.60102 (8)	0.56022 (7)	0.0169 (2)	
C8	0.17276 (9)	0.61556 (8)	0.64910 (6)	0.01590 (19)	
H8	0.103040	0.663851	0.660341	0.019*	
C9	0.23699 (9)	0.55986 (8)	0.72109 (6)	0.01566 (19)	
C10	0.12969 (10)	0.66135 (9)	0.83472 (7)	0.0217 (2)	
H10A	0.039594	0.656240	0.806104	0.026*	
H10B	0.169956	0.729787	0.814519	0.026*	
C11	0.12720 (12)	0.65932 (10)	0.93669 (7)	0.0291 (3)	
H11A	0.077430	0.722042	0.956894	0.044*	
H11B	0.217077	0.662715	0.964005	0.044*	
H11C	0.085471	0.591875	0.955557	0.044*	
C12	0.14577 (10)	0.66574 (9)	0.48019 (7)	0.0206 (2)	
C13	0.0172 (2)	0.7215 (2)	0.50405 (16)	0.0310 (5)	0.760 (6)
H13A	0.036459	0.777122	0.550703	0.047*	0.760 (6)
H13B	−0.042303	0.666985	0.527143	0.047*	0.760 (6)
H13C	−0.024326	0.755804	0.449467	0.047*	0.760 (6)
C14	0.1107 (3)	0.5888 (2)	0.39926 (14)	0.0369 (6)	0.760 (6)
H14A	0.068743	0.630696	0.348806	0.055*	0.760 (6)
H14B	0.050452	0.532275	0.418034	0.055*	0.760 (6)
H14C	0.190785	0.554669	0.379613	0.055*	0.760 (6)
C15	0.2408 (2)	0.7526 (2)	0.4503 (2)	0.0432 (7)	0.760 (6)
H15A	0.262925	0.802228	0.501104	0.065*	0.760 (6)
H15B	0.199701	0.793832	0.399145	0.065*	0.760 (6)
H15C	0.320808	0.717575	0.431530	0.065*	0.760 (6)
C13'	0.0046 (8)	0.6850 (7)	0.4909 (6)	0.0310 (5)	0.240 (6)
H13D	−0.006452	0.720457	0.549469	0.047*	0.240 (6)
H13E	−0.031072	0.732077	0.441575	0.047*	0.240 (6)
H13F	−0.042164	0.615282	0.488675	0.047*	0.240 (6)
C14'	0.1623 (10)	0.6123 (7)	0.3918 (5)	0.0369 (6)	0.240 (6)
H14D	0.255669	0.599493	0.384674	0.055*	0.240 (6)
H14E	0.115526	0.542548	0.389599	0.055*	0.240 (6)
H14F	0.126618	0.659343	0.342499	0.055*	0.240 (6)
C15'	0.2199 (9)	0.7761 (7)	0.4833 (7)	0.0432 (7)	0.240 (6)
H15D	0.209156	0.811794	0.541782	0.065*	0.240 (6)
H15E	0.313238	0.763249	0.476038	0.065*	0.240 (6)
H15F	0.184120	0.823022	0.433886	0.065*	0.240 (6)
C16	0.74702 (9)	0.34330 (8)	0.82783 (6)	0.0171 (2)	
C17	0.76462 (10)	0.23134 (9)	0.81993 (7)	0.0193 (2)	
C18	0.88612 (10)	0.18259 (9)	0.82380 (7)	0.0235 (2)	
H18	0.894197	0.105784	0.818617	0.028*	
C19	0.9963 (1)	0.24799 (10)	0.83543 (7)	0.0240 (2)	
H19	1.080842	0.215592	0.838175	0.029*	
C20	0.9849 (1)	0.36007 (10)	0.84310 (7)	0.0225 (2)	
H20	1.060505	0.404970	0.850461	0.027*	
C21	0.86114 (10)	0.40459 (9)	0.83979 (7)	0.0195 (2)	

### 4-(4-tert-Butyl-2-ethoxyphenyl)-2-(2,6-difluorophenyl)-4,5-dihydro-1,3-oxazole Atomic displacement parameters (Å^2^)

**Table:**

	*U*^11^	*U*^22^	*U*^33^	*U*^12^	*U*^13^	*U*^23^
F1	0.0192 (3)	0.0200 (3)	0.0382 (4)	−0.0005 (2)	−0.0007 (3)	−0.0033 (3)
F2	0.0228 (3)	0.0206 (3)	0.0420 (4)	−0.0021 (3)	0.0004 (3)	0.0023 (3)
O1	0.0189 (4)	0.0261 (4)	0.0202 (4)	0.0054 (3)	−0.0027 (3)	−0.0046 (3)
O2	0.0205 (4)	0.0233 (4)	0.0140 (3)	0.0068 (3)	0.0029 (3)	0.0019 (3)
N1	0.0176 (4)	0.0184 (4)	0.0219 (4)	0.0032 (3)	−0.0019 (3)	−0.0002 (3)
C1	0.0177 (5)	0.0158 (5)	0.0160 (4)	0.0004 (4)	0.0015 (4)	0.0016 (4)
C2	0.0182 (5)	0.0225 (5)	0.0200 (5)	0.0051 (4)	−0.0018 (4)	−0.0006 (4)
C3	0.0156 (5)	0.0173 (5)	0.0205 (5)	0.0015 (4)	0.0004 (4)	0.0005 (4)
C4	0.0148 (4)	0.0173 (5)	0.0172 (5)	−0.0007 (4)	−0.0011 (4)	−0.0001 (4)
C5	0.0173 (5)	0.0205 (5)	0.0200 (5)	0.0024 (4)	0.0011 (4)	−0.0032 (4)
C6	0.0212 (5)	0.0243 (5)	0.0152 (5)	−0.0001 (4)	0.0024 (4)	−0.0021 (4)
C7	0.0172 (5)	0.0172 (5)	0.0160 (5)	−0.0034 (4)	−0.0013 (4)	0.0007 (4)
C8	0.0136 (4)	0.0164 (5)	0.0176 (5)	−0.0001 (4)	0.0005 (3)	−0.0002 (4)
C9	0.0150 (4)	0.0174 (5)	0.0146 (4)	−0.0018 (4)	0.0013 (3)	−0.0006 (4)
C10	0.0229 (5)	0.0235 (5)	0.0191 (5)	0.0074 (4)	0.0035 (4)	−0.0002 (4)
C11	0.0345 (6)	0.0349 (6)	0.0184 (5)	0.0077 (5)	0.0050 (4)	−0.0024 (5)
C12	0.0227 (5)	0.0229 (5)	0.0161 (5)	0.0010 (4)	−0.0002 (4)	0.0035 (4)
C13	0.0370 (9)	0.0374 (15)	0.0184 (9)	0.0178 (11)	−0.0005 (7)	0.0035 (9)
C14	0.0525 (17)	0.0355 (11)	0.0205 (7)	0.0085 (11)	−0.0136 (10)	−0.0036 (7)
C15	0.0380 (11)	0.0437 (13)	0.0466 (17)	−0.0104 (9)	−0.0072 (10)	0.0282 (12)
C13'	0.0370 (9)	0.0374 (15)	0.0184 (9)	0.0178 (11)	−0.0005 (7)	0.0035 (9)
C14'	0.0525 (17)	0.0355 (11)	0.0205 (7)	0.0085 (11)	−0.0136 (10)	−0.0036 (7)
C15'	0.0380 (11)	0.0437 (13)	0.0466 (17)	−0.0104 (9)	−0.0072 (10)	0.0282 (12)
C16	0.0160 (5)	0.0215 (5)	0.0135 (4)	0.0018 (4)	−0.0002 (3)	0.0014 (4)
C17	0.0165 (5)	0.0227 (5)	0.0185 (5)	0.0001 (4)	−0.0003 (4)	−0.0006 (4)
C18	0.0228 (5)	0.0238 (5)	0.0239 (5)	0.0064 (4)	0.0010 (4)	−0.0005 (4)
C19	0.0169 (5)	0.0348 (6)	0.0203 (5)	0.0078 (4)	0.0009 (4)	0.0012 (4)
C20	0.0163 (5)	0.0320 (6)	0.0191 (5)	−0.0016 (4)	0.0004 (4)	0.0021 (4)
C21	0.0210 (5)	0.0199 (5)	0.0174 (5)	0.0008 (4)	0.0005 (4)	0.0023 (4)

### 4-(4-tert-Butyl-2-ethoxyphenyl)-2-(2,6-difluorophenyl)-4,5-dihydro-1,3-oxazole Geometric parameters (Å, º)

**Table:**

F1—C17	1.3458 (12)	C12—C15	1.527 (2)
F2—C21	1.3475 (12)	C12—C13	1.543 (2)
O1—C1	1.3598 (12)	C12—C14	1.545 (2)
O1—C2	1.4566 (12)	C12—C15'	1.552 (8)
O2—C9	1.3719 (11)	C13—H13A	0.9800
O2—C10	1.4370 (12)	C13—H13B	0.9800
N1—C1	1.2663 (13)	C13—H13C	0.9800
N1—C3	1.4775 (12)	C14—H14A	0.9800
C1—C16	1.4777 (13)	C14—H14B	0.9800
C2—C3	1.5425 (14)	C14—H14C	0.9800
C2—H2A	0.9900	C15—H15A	0.9800
C2—H2B	0.9900	C15—H15B	0.9800
C3—C4	1.5128 (13)	C15—H15C	0.9800
C3—H3	1.0000	C13'—H13D	0.9800
C4—C5	1.3878 (14)	C13'—H13E	0.9800
C4—C9	1.3979 (13)	C13'—H13F	0.9800
C5—C6	1.3913 (14)	C14'—H14D	0.9800
C5—H5	0.9500	C14'—H14E	0.9800
C6—C7	1.3946 (14)	C14'—H14F	0.9800
C6—H6	0.9500	C15'—H15D	0.9800
C7—C8	1.3994 (13)	C15'—H15E	0.9800
C7—C12	1.5343 (14)	C15'—H15F	0.9800
C8—C9	1.3910 (13)	C16—C21	1.3900 (14)
C8—H8	0.9500	C16—C17	1.3921 (14)
C10—C11	1.5051 (14)	C17—C18	1.3768 (14)
C10—H10A	0.9900	C18—C19	1.3850 (16)
C10—H10B	0.9900	C18—H18	0.9500
C11—H11A	0.9800	C19—C20	1.3861 (16)
C11—H11B	0.9800	C19—H19	0.9500
C11—H11C	0.9800	C20—C21	1.3764 (14)
C12—C14'	1.479 (7)	C20—H20	0.9500
C12—C13'	1.482 (8)		
			
C1—O1—C2	105.02 (7)	C14'—C12—C15'	109.3 (4)
C9—O2—C10	118.04 (8)	C13'—C12—C15'	109.6 (5)
C1—N1—C3	106.35 (8)	C7—C12—C15'	104.2 (3)
N1—C1—O1	119.37 (9)	C12—C13—H13A	109.5
N1—C1—C16	124.93 (9)	C12—C13—H13B	109.5
O1—C1—C16	115.68 (8)	H13A—C13—H13B	109.5
O1—C2—C3	103.82 (8)	C12—C13—H13C	109.5
O1—C2—H2A	111.0	H13A—C13—H13C	109.5
C3—C2—H2A	111.0	H13B—C13—H13C	109.5
O1—C2—H2B	111.0	C12—C14—H14A	109.5
C3—C2—H2B	111.0	C12—C14—H14B	109.5
H2A—C2—H2B	109.0	H14A—C14—H14B	109.5
N1—C3—C4	112.14 (8)	C12—C14—H14C	109.5
N1—C3—C2	104.05 (8)	H14A—C14—H14C	109.5
C4—C3—C2	114.55 (8)	H14B—C14—H14C	109.5
N1—C3—H3	108.6	C12—C15—H15A	109.5
C4—C3—H3	108.6	C12—C15—H15B	109.5
C2—C3—H3	108.6	H15A—C15—H15B	109.5
C5—C4—C9	117.71 (9)	C12—C15—H15C	109.5
C5—C4—C3	123.36 (9)	H15A—C15—H15C	109.5
C9—C4—C3	118.93 (8)	H15B—C15—H15C	109.5
C4—C5—C6	121.06 (9)	C12—C13'—H13D	109.5
C4—C5—H5	119.5	C12—C13'—H13E	109.5
C6—C5—H5	119.5	H13D—C13'—H13E	109.5
C5—C6—C7	121.36 (9)	C12—C13'—H13F	109.5
C5—C6—H6	119.3	H13D—C13'—H13F	109.5
C7—C6—H6	119.3	H13E—C13'—H13F	109.5
C6—C7—C8	117.80 (9)	C12—C14'—H14D	109.5
C6—C7—C12	120.52 (9)	C12—C14'—H14E	109.5
C8—C7—C12	121.64 (9)	H14D—C14'—H14E	109.5
C9—C8—C7	120.51 (9)	C12—C14'—H14F	109.5
C9—C8—H8	119.7	H14D—C14'—H14F	109.5
C7—C8—H8	119.7	H14E—C14'—H14F	109.5
O2—C9—C8	124.17 (9)	C12—C15'—H15D	109.5
O2—C9—C4	114.27 (8)	C12—C15'—H15E	109.5
C8—C9—C4	121.56 (9)	H15D—C15'—H15E	109.5
O2—C10—C11	106.58 (8)	C12—C15'—H15F	109.5
O2—C10—H10A	110.4	H15D—C15'—H15F	109.5
C11—C10—H10A	110.4	H15E—C15'—H15F	109.5
O2—C10—H10B	110.4	C21—C16—C17	115.66 (9)
C11—C10—H10B	110.4	C21—C16—C1	122.74 (9)
H10A—C10—H10B	108.6	C17—C16—C1	121.60 (9)
C10—C11—H11A	109.5	F1—C17—C18	118.17 (9)
C10—C11—H11B	109.5	F1—C17—C16	118.65 (9)
H11A—C11—H11B	109.5	C18—C17—C16	123.16 (10)
C10—C11—H11C	109.5	C17—C18—C19	118.53 (10)
H11A—C11—H11C	109.5	C17—C18—H18	120.7
H11B—C11—H11C	109.5	C19—C18—H18	120.7
C14'—C12—C13'	109.4 (4)	C18—C19—C20	120.89 (10)
C14'—C12—C7	112.3 (3)	C18—C19—H19	119.6
C13'—C12—C7	111.9 (4)	C20—C19—H19	119.6
C15—C12—C7	109.55 (12)	C21—C20—C19	118.25 (10)
C15—C12—C13	108.88 (14)	C21—C20—H20	120.9
C7—C12—C13	112.54 (11)	C19—C20—H20	120.9
C15—C12—C14	108.92 (14)	F2—C21—C20	118.57 (9)
C7—C12—C14	110.08 (11)	F2—C21—C16	117.92 (9)
C13—C12—C14	106.79 (13)	C20—C21—C16	123.5 (1)
			
C3—N1—C1—O1	−2.09 (12)	C8—C7—C12—C14'	−159.2 (4)
C3—N1—C1—C16	176.16 (9)	C6—C7—C12—C13'	147.0 (4)
C2—O1—C1—N1	−5.81 (12)	C8—C7—C12—C13'	−35.6 (4)
C2—O1—C1—C16	175.78 (8)	C6—C7—C12—C15	−71.21 (19)
C1—O1—C2—C3	10.39 (10)	C8—C7—C12—C15	106.10 (18)
C1—N1—C3—C4	132.90 (9)	C6—C7—C12—C13	167.52 (15)
C1—N1—C3—C2	8.55 (10)	C8—C7—C12—C13	−15.16 (18)
O1—C2—C3—N1	−11.53 (10)	C6—C7—C12—C14	48.53 (18)
O1—C2—C3—C4	−134.32 (8)	C8—C7—C12—C14	−134.15 (16)
N1—C3—C4—C5	6.09 (14)	C6—C7—C12—C15'	−94.6 (4)
C2—C3—C4—C5	124.39 (11)	C8—C7—C12—C15'	82.7 (4)
N1—C3—C4—C9	−173.72 (8)	N1—C1—C16—C21	134.33 (11)
C2—C3—C4—C9	−55.42 (12)	O1—C1—C16—C21	−47.36 (13)
C9—C4—C5—C6	0.95 (15)	N1—C1—C16—C17	−45.09 (15)
C3—C4—C5—C6	−178.86 (9)	O1—C1—C16—C17	133.22 (10)
C4—C5—C6—C7	−0.24 (16)	C21—C16—C17—F1	178.67 (9)
C5—C6—C7—C8	−0.54 (15)	C1—C16—C17—F1	−1.88 (14)
C5—C6—C7—C12	176.88 (9)	C21—C16—C17—C18	0.21 (15)
C6—C7—C8—C9	0.59 (14)	C1—C16—C17—C18	179.67 (9)
C12—C7—C8—C9	−176.80 (9)	F1—C17—C18—C19	−178.97 (9)
C10—O2—C9—C8	−17.70 (14)	C16—C17—C18—C19	−0.50 (16)
C10—O2—C9—C4	162.47 (9)	C17—C18—C19—C20	0.06 (16)
C7—C8—C9—O2	−179.68 (9)	C18—C19—C20—C21	0.64 (16)
C7—C8—C9—C4	0.14 (15)	C19—C20—C21—F2	−179.62 (9)
C5—C4—C9—O2	178.92 (9)	C19—C20—C21—C16	−0.97 (16)
C3—C4—C9—O2	−1.26 (13)	C17—C16—C21—F2	179.21 (9)
C5—C4—C9—C8	−0.91 (15)	C1—C16—C21—F2	−0.24 (14)
C3—C4—C9—C8	178.91 (9)	C17—C16—C21—C20	0.55 (15)
C9—O2—C10—C11	−172.14 (9)	C1—C16—C21—C20	−178.90 (9)
C6—C7—C12—C14'	23.5 (4)		

### 4-(4-tert-Butyl-2-ethoxyphenyl)-2-(2,6-difluorophenyl)-4,5-dihydro-1,3-oxazole Hydrogen-bond geometry (Å, º)

**Table:**

*D*—H···*A*	*D*—H	H···*A*	*D*···*A*	*D*—H···*A*
C10—H10*A*···F1^i^	0.99	2.57	3.5211 (13)	162
C20—H20···O2^ii^	0.95	2.60	3.4766 (13)	154

Symmetry codes: (i) −*x*+1/2, *y*+1/2, −*z*+3/2; (ii) *x*+1, *y*, *z*.
